# Tumor-derived miR-130b-3p induces cancer-associated fibroblast activation by targeting SPIN90 in luminal A breast cancer

**DOI:** 10.1038/s41389-022-00422-6

**Published:** 2022-08-10

**Authors:** Suyeon Ahn, Ahreum Kwon, Yun Hyun Huh, Sangmyung Rhee, Woo Keun Song

**Affiliations:** 1 Cell Logistics Research Center, School of Life Sciences, Gwangju Institute of Science and Technology, Gwangju, 61005 Republic of Korea; 2 School of Life Sciences, Gwangju Institute of Science and Technology, Gwangju, 61005 Republic of Korea; 3 Department of Life Science, Chung-Ang University, Seoul, 06974 Republic of Korea

**Keywords:** Breast cancer, Non-coding RNAs

## Abstract

Cancer-associated fibroblasts (CAFs) in the tumor microenvironment (TME) interact closely with cancer cells to promote tumor development. Downregulation of SPIN90 in CAFs has been reported to facilitate breast cancer progression, but the underlying mechanism has not been elucidated. Here, we demonstrate that miR-130b-3p directly downregulates SPIN90 in stromal fibroblasts, leading to their differentiation into CAFs. As the decrease of SPIN90 in CAFs was shown to be more prominent in estrogen receptor (ER)-positive breast tumors in this study, miR-130b-3p was selected by bioinformatics analysis of data from patients with ER-positive breast cancer. Ectopic expression of miR-130b-3p in fibroblasts accelerated their differentiation to CAFs that promote cancer cell motility; this was associated with SPIN90 downregulation. We also found that miR-130b-3p was generated in luminal A-type cancer cells and activated fibroblasts after being secreted via exosomes from cancer cells. Finally, miR-130b-3p increased in SPIN90-downregulated tumor stroma of luminal A breast cancer patients and MCF7 cell-xenograft model mice. Our data demonstrate that miR-130b-3p is a key modulator that downregulates SPIN90 in breast CAFs. The inverse correlation between miR-130b-3p and SPIN90 in tumor stroma suggests that the miR-130b-3p/SPIN90 axis is clinically significant for CAF activation during breast cancer progression.

## Introduction

Breast cancer is the most common cancer and the second leading cause of cancer death in women [1]. It may be classified into four molecular subtypes based on its surface receptor expression: luminal A, luminal B, Her2-enriched, and triple-negative [2]. Estrogen receptor (ER), progesterone receptor (PR), and human epidermal growth factor receptor 2 (HER2) are involved in determining the subtypes [3], and more than 70% of breast tumors are found to express ER [4]. Although the use of adjuvant endocrine therapy and surgery has considerably improved survival rates, 20–40% of patients develop distant metastasis. In tumor metastasis, the crosstalk between cancer cells and their microenvironment is important [5, 6]. The tumor microenvironment (TME), which is composed of fibroblasts, immune cells, endothelial cells, and extracellular matrix (ECM), plays a pivotal role in breast cancer growth and invasion [7].

A type of activated fibroblasts in cancer tissues, termed cancer-associated fibroblasts (CAFs), represents the most abundant cell type in the TME [8]. CAFs contribute to cancer progression by secreting matrix-crosslinking enzymes [9], matrix components used to remodel the ECM [10], and growth factors that modulate tumor growth and chemotherapy resistance [11–13]. Myofibroblastic CAFs are characterized by elevated expression of alpha-smooth muscle actin (α-SMA) [14], fibroblast activation protein (FAP) [15], and connective tissue growth factor (CTGF) [16, 17]. Differentiation of stromal fibroblasts into CAFs is known to be induced by paracrine signals from cancer cells [11], direct-contact signals such as Notch [18], and physical alterations in the ECM [19]. CAFs are considered to represent a therapeutic target for breast cancer [20]. Hence, further mechanistic studies of CAF activation are needed.

In our previous work, we revealed that SPIN90 (SH3 protein interacting with Nck, 90 kDa) [21] is significantly decreased in the tumor stroma of breast cancer patients and that SPIN90-knockout (KO) mouse embryonic fibroblasts (MEFs) showed a more myofibroblastic CAF phenotype than wild-type MEFs [22]. We further showed that breast cancer progression in a mouse model was markedly enhanced from an early stage after co-injection of SPIN90 KO MEFs plus tumor cells, whereas co-injection of SPIN90-rescued MEFs compensated for this effect on tumor development [22]. These data collectively suggest that SPIN90 depletion in stromal fibroblasts leads to CAF activation, breast cancer growth, and invasion. Further work is needed to elucidate the molecular mechanisms underlying the downregulation of SPIN90 in breast cancer TME.

Because genetic mutations in CAFs are rare compared to the frequent changes seen in cancer cells, alternative methods of cancer-related gene regulation may occur in CAFs [23, 24]. microRNAs (miRNAs) have emerged as key post-transcriptional regulators in cancer biology [25]. They are small non-coding RNAs that degrade or inhibit the translation of target mRNAs by directly binding to their 3’ untranslated regions (UTR) [26]. miRNAs are known to be poorly controlled when cancer develops [27], and they can be differentially expressed in accordance with the pathological subtype of breast cancer [28]. Several studies have revealed that dysregulated miRNAs secreted from tumor tissues perform paracrine functions in the TME [29] and can reprogram normal fibroblasts to CAFs [30]. In pancreatic cancer, miR-155 in cancer cell-derived microvesicles are known to induce CAF activation by targeting TP53INP1 [31]. However, the detailed mechanisms through which miRNAs act as an “on” switch for breast CAF activation have not been fully elucidated.

In this study, we investigated whether a SPIN90-targeting miRNA could represent a mechanism for stromal fibroblast activation in breast cancer. A candidate miRNA was selected using bioinformatics and biomolecular analysis, and the efficacy of SPIN90 downregulation and CAF activation was evaluated. We examined the expression of the selected miRNA in various human breast cancer tissues to explore the primary secreting cell types. Furthermore, we confirmed the clinical significance of this miRNA as a SPIN90 repressor in breast cancer by analyzing in vivo orthotopic breast cancer model mice and human breast cancer patient data. Taken together, our results lead us to propose the selected miRNA as a novel regulator of SPIN90 during CAF differentiation in the breast TME.

## Results

### SPIN90 depletion in CAFs is more prominent in ER-positive breast cancer tissues and has more clinical relevance in ER-positive breast cancer patients

**Fig. 1:**
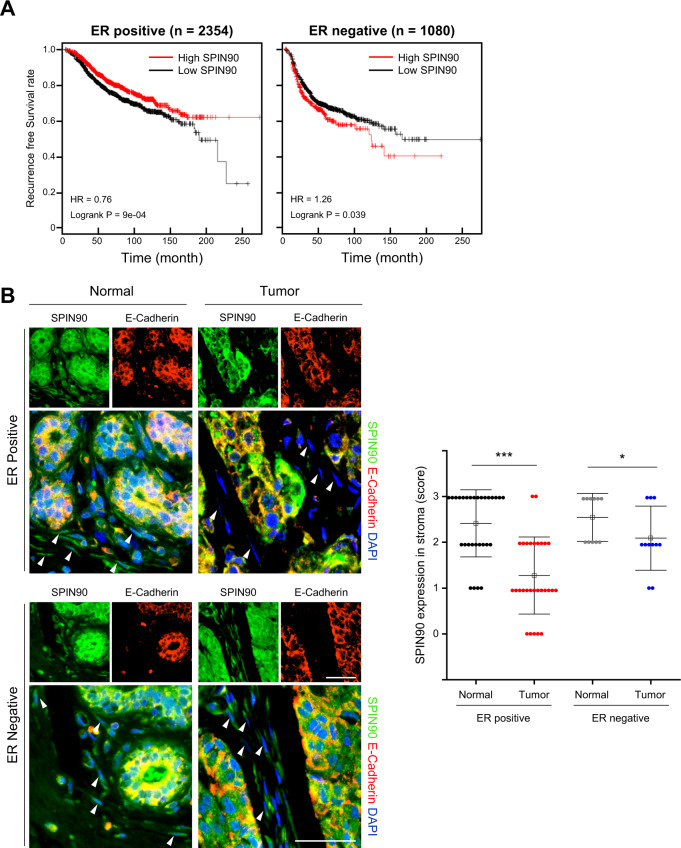
SPIN90 is significantly downregulated in CAFs of ER-positive breast cancer patients. **A** KM plots of recurrence-free survival in ER-positive (*n* = 2354) and ER-negative (*n* = 1080) breast cancer patients according to *Spin90* level. Outlier data were removed for quality control. HR hazard ratio. **B** Representative IHC images showing expression of SPIN90 (green) and the epithelial cell marker E-cadherin (red) in breast cancers and paired normal stroma from ER-positive (*n* = 29) and ER-negative (*n* = 11) samples in a tumor microarray (BR804b). The white arrows indicate stromal fibroblasts. The graph shows the relative intensity score of SPIN90 in stroma as follows: 0 = negative, 1 = weak, 2 = moderate, 3 = strong. The images were scanned with an Olympus research slide scanner and analyzed using the Olympus cellSens and ImageJ software packages. Scale bar, 50 μm. All data are presented as mean ± standard deviation. *p ≤ 0.05; ***p ≤ 0.001 (Student’s *t*-test).

Before investigating miRNAs that could modulate SPIN90, we verified the impact of SPIN90 downregulation in human breast cancer cases. As ER is considered to be an important factor in the progression and diagnosis of most breast cancers, the relationship between SPIN90 expression and patient survival was assessed according to the presence or absence of ER. Kaplan–Meier (KM; https://kmplot.com/analysis/; Affymetrix ID: 216116) analysis revealed that a lower SPIN90 level in tumor tissues is associated with shorter survival rates in ER-positive patients (*n* = 2354), not in ER-negative patients (*n* = 1080) (Fig. 1A). This suggests that SPIN90 depletion in tumor tissues is more clinically relevant in ER-positive breast cancer.

Next, immunostaining was performed to compare SPIN90 expression patterns in a commercially available array that included 29 ER-positive and 11 ER-negative human breast cancers and paired normal tissues. Breast epithelial cells were distinguished from stromal cells by staining with antibodies to the epithelial cell marker E-cadherin. The level of SPIN90 in normal epithelial cells and tumor cells was unaffected by ER status, but the tumor stroma showed a significant reduction of SPIN90-positive cells compared to normal stroma in both ER-positive and -negative types. Among them, the decreasing trend of SPIN90 in CAFs compared to normal fibroblasts was more pronounced in ER-positive tissues (Fig. 1B). The intensity of SPIN90 was classified according to the criteria presented in Supplementary Fig. S1A. CAFs in 62% of the ER-positive tumor tissues showed a SPIN90 intensity score <2, while only 18% of the ER-negative tumor tissues had an intensity score <2. Overall, these results (Fig. 1) demonstrate that SPIN90 expression is lower in CAFs around ER-positive breast cancer, and the decrease of SPIN90 is more strongly correlated with poor survival rates in ER-positive patients than in ER-negative patients.

### miR-130b-3p downregulates SPIN90 mRNA and protein expression by directly binding to the *Spin90* mRNA 3’UTR

**Fig. 2:**
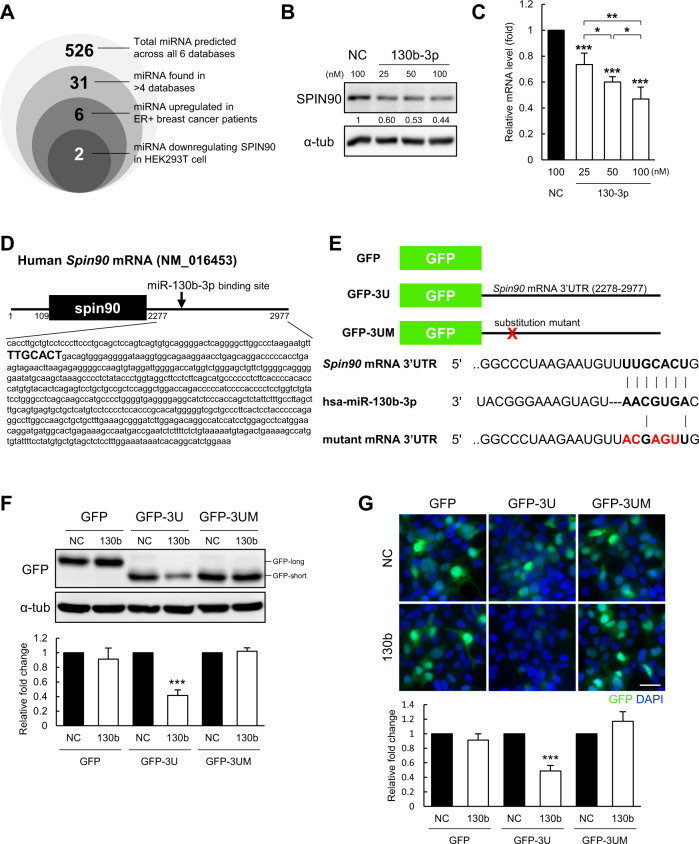
miR-130b-3p inhibits SPIN90 expression by complementary binding. **A** Venn diagram showing the process used to narrow down candidate SPIN90-targeting miRNAs through in silico and in vitro analyses. **B** Western blot indicating the expression of SPIN90 in HEK-293T cells transfected with miR-130b-3p mimic or negative control (*n* = 3). Relative protein levels were normalized to the expression of α-tub. **C** The *Spin90* mRNA level was quantified by RT-qPCR assay in HEK-293T cells transfected with miR-130b-3p mimic or negative control (*n* = 3). The relative mRNA level was normalized to the expression of *Gapdh*. **D** Sequence information of the *Spin90* mRNA indicating the predicted binding site (bolded sequences) for miR-130b-3p. **E** Reporter gene constructs. GFP-3U indicates a GFP vector inserted with the 3’UTR of the *Spin90* mRNA, and GFP-3UM refers to a mutant construct lacking the binding site for miR-130b-3p. Bolded sequences, seed region. Red sequences, mutated region. **F** Reporter gene assay examined by Western blotting in HEK-293T cells co-transfected with reporter constructs and miRNAs (*n* = 3). GFP protein size depends on the location of the stop codon in each reporter gene construct. **G** Immunofluorescence assays of reporter gene expression in HEK-293T cells (*n* = 3). Scale bar, 50 μm. All data are reported as mean ± standard deviation. ***p ≤ 0.001 by Student’s *t*-tests. NC negative control of the miRNA mimic; 130b and 130b-3p, miR-130b-3p mimic.

To seek miRNAs that might contribute to governing SPIN90 within the CAFs of ER-positive patients, we carried out bioinformatics analysis. We searched for potential sequence matches indicating the possibility of direct binding in six different prediction databases: TargetScan, miRmap, miRWalk, miRsystem, Tarbase, and miRDB. We then selected miRNAs that were identified in at least four of the databases (31 miRNAs) and subjected them to KM plot analysis in ER-positive patients. Six miRNAs found to be highly expressed in ER-positive patients with lower survival rates were transfected into HEK-293T cells to assess their ability to inhibit SPIN90 protein expression (Fig. 2A). The results revealed that ectopic expression of a synthetic miR-130b-3p mimic (hsa-miR-130b-3p) significantly and concentration-dependently downregulated SPIN90 at the protein and mRNA levels (Fig. 2B, C). Another candidate, miR-6751–5p, also reduced the protein and mRNA expression levels of SPIN90 (Supplementary Fig. S1B, C). However, we excluded this candidate from further analysis because its endogenous expression in vitro was negligible (Ct value > 33; Supplementary Fig. S1D).

We next ascertained whether miR-130b-3p directly targets SPIN90 expression. The TargetScan program (http://www.targetscan.org/) was used to identify the seed region of miR-130b-3p, which was predicted to correspond to the nt 75–81 of the *Spin90* mRNA 3’UTR (Fig. 2D). The 3’UTR sequence of the *Spin90* mRNA transcript was confirmed in the NCBI Gene database (Accession No. NM_016453). We constructed a GFP reporter gene containing the 3’UTR region of the *Spin90* mRNA (GFP-3U) or a mutant (GFP-3UM) with substitution of five of the seven nucleotides in the seed region (Fig. 2E). Overexpression of miR-130b-3p mimic significantly reduced the protein expression of the GFP reporter gene containing the 3’UTR of *Spin90* mRNA, but not the control GFP or mutant reporters (Fig. 2F). Moreover, miR-130b-3p was found to bind the *Spin90* 3’UTR sequence to uniformly suppress the fluorescence activity of GFP (Fig. 2G). It was additionally confirmed that miR-130b-3p did not exhibit any off-target effect that regulates the expression of proteins other than SPIN90 (Supplementary Fig. S1E). Overall, these results indicate that miR-130b-3p suppresses SPIN90 expression by binding a complementary region in the 3’UTR region of the *Spin90* mRNA.

### Fibroblasts activated by miR-130b-3p exhibit characteristics of cancer-promoting myofibroblastic CAFs

**Fig. 3:**
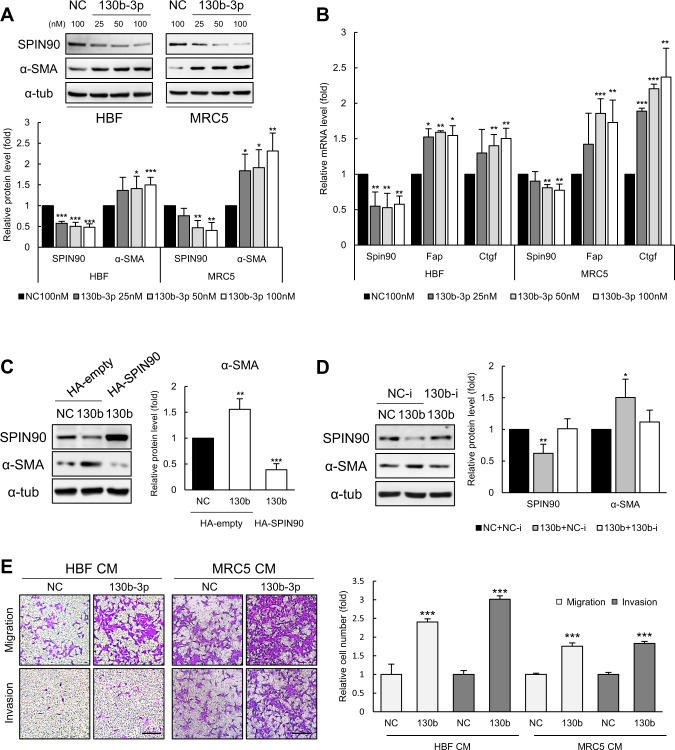
miR-130b-3p activates fibroblasts to exhibit CAF properties. **A** Fibroblast activation ability of miR-130b-3p, as assessed by Western blot analysis. miR-130b-3p was transfected to HBFs and MRC5 cells at 25, 50, and 100 nM for 48 h, and histograms were normalized to the expression in the normal control (NC; black bar) of each group (*n* = 3). **B** Relative mRNA expression of *Spin90* and myofibroblast markers, as examined by RT-qPCR (*n* = 3). **C** Recovery of SPIN90 expression in miR-130b-3p mimic transfected HBFs (*n* = 3). miRNAs were applied for 24 h, cells were transfected with HA or HA-SPIN90 DNA vector for 24 h, and lysates were collected. **D** Inhibition of the effect of miR-130b-3p on fibroblasts by transient expression of QIAGEN LNA miR-130b-3p power inhibitor. The oligonucleotides were co-transfected to HBFs with miRNA mimics for 48 h (*n* = 3). **E** Migration and invasion assay of MCF7 cells. CM from miRNA mimic-treated fibroblasts was added to the lower chamber. MCF7 cells were seeded in matrigel-coated or -uncoated upper chambers. Scale bar, 100 μm. All data are presented as mean ± standard deviation. *p ≤ 0.05; **p ≤ 0.01; ***p ≤ 0.001 (Student’s *t*-test). NC negative control of miRNA mimic; 130b and 130b-3p, miR-130b-3p mimic; NC-i, inhibitor of negative control; 130b-i, inhibitor of miR-130b-3p.

We previously showed that SPIN90 depletion leads to fibroblast activation, which contributes to breast cancer tumorigenesis [22, 32, 33]. Thus, we herein examined whether overexpression of the SPIN90-targeting miRNA, miR-130b-3p, could induce fibroblast activation. Transfection of miR-130b-3p mimic to human breast skin fibroblasts (HBFs) or MRC5, human lung fibroblast cell line, caused SPIN90 downregulation and significantly increased the expression of myofibroblast markers, including α-SMA, *Fap*, and *Ctgf* (Fig. 3A, B). The upregulation of α-SMA by miR-130b-3p mimic transfection was rescued by ectopic expression of SPIN90 (Fig. 3C). Similarly, treatment with a miR-130b-3p inhibitor downregulated the increased expression of α-SMA in miR-130b-3p mimic-transfected cells (Fig. 3D). These findings suggest that miR-130b-3p promotes fibroblasts activation by directly targeting SPIN90.

Activated fibroblasts in the TME were reported to facilitate the motility of cancer cells by secreting growth factors or remodeling the ECM [20]. Thus, we next examined the effect of conditioned media (CM) obtained from miR-130b-3p mimic-transfected fibroblasts on cancer cell motility, using a Boyden chamber and wound-healing assay. As shown in Fig. 3E, CM of miR-130b-3p mimic-treated HBFs and MRC5 cells increased the migration and invasion of MCF7 cells, which were selected as a representative ER-positive breast cancer cell line. A scratch-wound-healing assay yielded similar results (Supplementary Fig. S2A). These data demonstrate that fibroblasts activated by the presence of miR-130b-3p in the TME have the potential to promote cancer cell migration and invasion.

### miR-130b-3p, expressed primarily in luminal A breast cancer cells, is transferred to fibroblasts via exosomes, reducing SPIN90 and activating fibroblasts

**Fig. 4:**
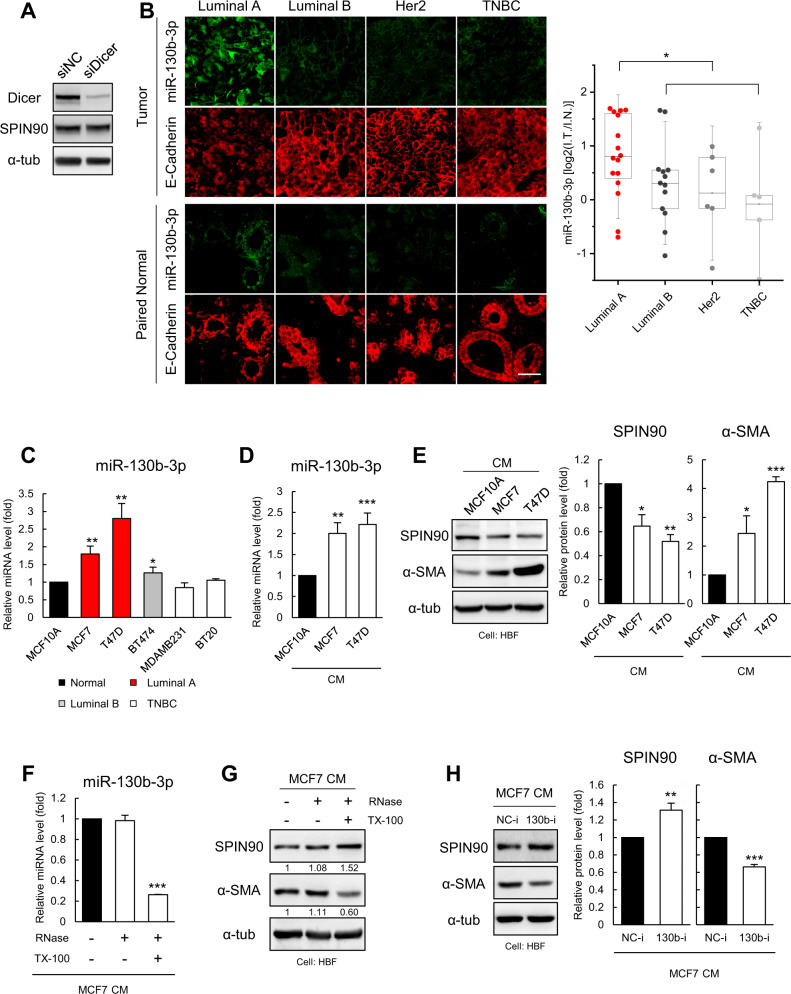
miR-130b-3p, which is secreted mostly from luminal A breast cancer, acts as a SPIN90 repressor in fibroblasts. **A** Dicer knockdown in HBF. Dicer and SPIN90 expression were detected by Western blotting. **B** Representative images showing expression of miR-130b-3p (green) and the epithelial cell marker E-cadherin (red) in tumor and paired normal tissues from a commercially available tumor microarray (BR804b, Luminal A (*n* = 16), Luminal B (*n* = 13), Her2-enriched (*n* = 6), TNBC (*n* = 5)). Graph indicates log ratio for the intensity of miR-130b-3p in tumor tissues relative to normal tissues. Scale bar, 50 μm. **C** RT-qPCR analysis quantified the expression of mature miR-130b-3p in cancer cell lines of various luminal types, compared to that in normal MCF10A cells (*n* = 3). The level of each miRNA was normalized to that of RNU6. **D** Relative expression of miR-130b-3p in CM enriched from luminal A cancer and normal cell lines, normalized by the average Ct level of miR-23a-3p, miR-191-5p, miR-425-5p, and miR-451a (*n* = 3). **E** Western blotting analysis for SPIN90 and α-SMA in HBFs incubated for 48 h in the indicated CM (*n* = 3). **F** Relative amounts of miR-130b-3p in MCF7 CM treated with RNase in the presence or absence of Triton X-100 (*n* = 3 each). MCF7-derived CM was incubated with 10 μg/mL RNase or 1% (v/v) Triton X-100 at 37 °C for 30 min. **G** Western blot analysis of HBF cells incubated with MCF7 CM in the presence or absence of RNase and/or Triton X-100. The expression of each protein was normalized relative to that of α-tub. Buffer in MCF7 CM was replaced using a centrifugal filter to minimize Triton X-100 cytotoxicity. **H** HBFs were treated with CM from MCF7 cells stably expressing an oligonucleotide inhibitor for miR-130b-3p (130b-i) or a negative control oligonucleotide (NC-i) (*n* = 3). All data are presented as mean ± standard deviation. *p ≤ 0.05; **p ≤ 0.01; ***p ≤ 0.001 (Student’s *t*-test).

Since it has been reported that the miRNAs released from cancer cells to the extracellular space are often delivered to constituent cells of the TME to regulate the genes of these recipient cells [34], we investigated the origin of the miR-130b-3p that acts to downregulate SPIN90 in fibroblasts. The siRNA-mediated downregulation of the crucial miRNA processor, Dicer, by ~90% in HBFs (Fig. 4A) did not affect the level of SPIN90 in these cells, suggesting that miR-130b-3p may not be endogenously expressed in fibroblasts [35].

Next, we quantified miR-130b-3p expression by in situ hybridization of a tumor and adjacent normal tissue microarray (BR804b) derived from human breast cancer patients. Breast normal and cancer cells were distinguished from stromal cells by staining with antibody to the epithelial marker E-cadherin. We found that 37.5% of luminal A-type breast tumor tissues showed more than 2-fold upregulation of miR-130b-3p compared to adjacent normal tissues, whereas only marginal expression of miR-130b-3p was observed in other types of breast cancer (Fig. 4B). Our RT-qPCR results also showed that the expression of miR-130b-3p was notably higher in luminal A cell lines, including MCF7 and T47D cells, compared to other groups (Fig. 4C). Expression of the precursor of miR-130b-3p was also highly increased in luminal A cell lines (Supplementary Fig. S2B). However, there was only minimal expression of miR-130b-3p and its precursor in HBF (Supplementary Fig. S2D). These findings suggest that miR-130b-3p is likely to be endogenously generated in cancer cells rather than fibroblasts.

To examine whether the miR-130b-3p generated in luminal A cell lines can be transferred to fibroblasts, CM from MCF7, T47D, and MCF10A cells were concentrated. RT-qPCR analysis showed that more miR-130b-3p was contained in CM derived from MCF7 and T47D cells than from MCF10A cells (Fig. 4D). Fibroblasts incubated with CM obtained from the luminal A cell lines exhibited downregulation of SPIN90 and upregulation of α-SMA (Fig. 4E, Supplementary Fig. S2C). miR-130b-3p in MCF7-derived CM was degraded by RNase in the presence, but not in the absence, of Triton X-100, suggesting that miR-130b-3p is packaged in and secreted from exosomes (Fig. 4F). Exosomes were collected from MCF10A and MCF7 CMs by ultracentrifugation, and MCF7 exosomes containing high concentrations of miR-130b-3p more readily promoted fibroblast activation than MCF10A exosomes (Supplementary Fig. S3D, E). None of MCF7 CM treated with RNase/Triton X-100, MCF7 130b-i CM, or exosomes obtained from MCF7 130b-i cells induced SPIN90 downregulation and fibroblasts activation (Fig. 4G, H, Supplementary Fig. S3J). Taken together, these results support the hypothesis that miR-130b-3p is secreted in the form of exosomes from luminal A breast cancer cells and regulates SPIN90 expression in fibroblasts. Direct inhibition of miR-130b-3p in MCF7-derived CM and inhibition of exosome formation by MCF7 cells did not reduce SPIN90 or increase α-SMA levels in fibroblasts (Supplementary Fig. S2E, H), indicating that miR-130b-3p in exosomes affects fibroblast activation.

### miR-130b-3p upregulation is observed in SPIN90-downregulated CAFs from xenograft model mice, and miR-130b-3p inhibition attenuates CAF development in vivo

**Fig. 5:**
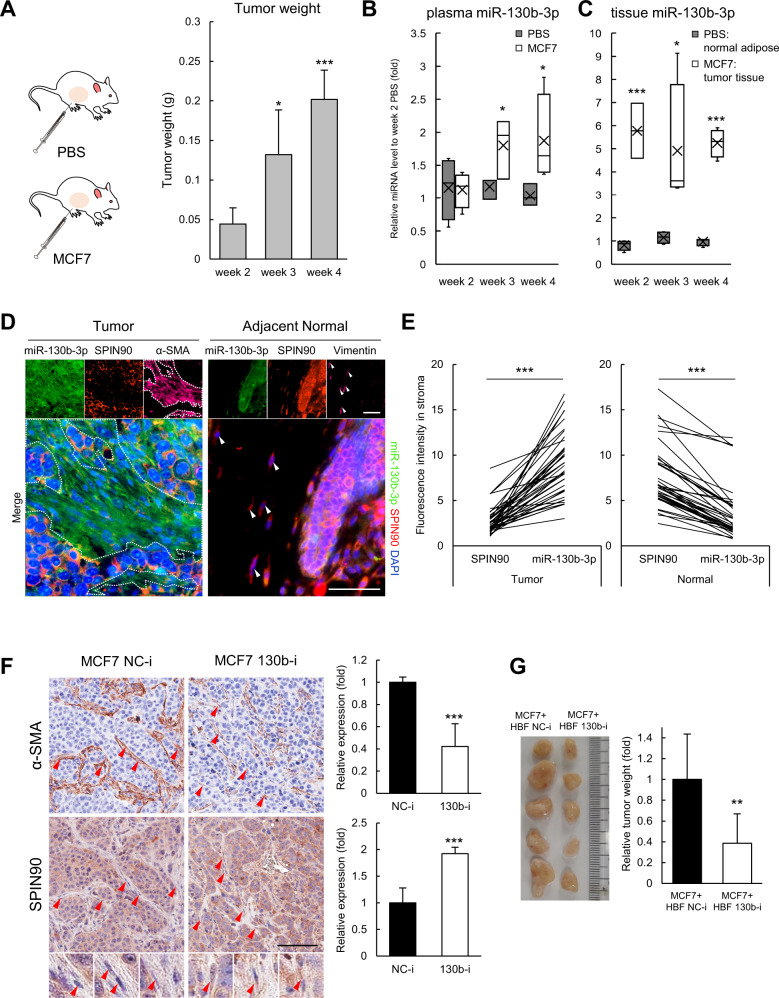
Expression pattern and role of miR-130b-3p in MCF7 xenografts of immune-deficient mice. **A** Mammary fat pad injection of MCF7 cells or PBS (negative control). The graph shows tumor growth in the MCF7-injected group. **B** Mouse plasma was collected at weeks 2, 3, and 4 (each group, *n* = 4) after tumor cell injection. miR-130b-3p in plasma was quantified through RT-qPCR assay and normalized by the average Ct levels of miR-23a-3p, miR-191-5p, miR-425-5p, and miR-451a. **C** Tumors from the MCF7 group and normal fat pads from the PBS group were isolated at weeks 2, 3, and 4. Tissues were homogenized in QIAzol lysis reagent and the miR-130b-3p level was examined. miRNAs from tissues were normalized by the expression of RNU6. **D**, Co-staining of miR-130b-3p (green) and SPIN90 (red) and the fibroblast markers α-SMA for CAFs and Vimentin for normal fibroblasts (magenta) in tumor and adjacent normal tissues (*n* = 4 per group) from the MCF7 group. White dotted lines and arrows, stromal fibroblasts. The merged images show samples co-stained for miR-130b-3p and SPIN90, and with DAPI. Scale bar, 25 μm. **E** Expression pattern of miR-130b-3p and SPIN90 in tumor and normal tissues. The fluorescent intensity in the stroma was analyzed by the ImageJ software. **F** Representative IHC images of xenografted tumors derived from MCF7 NC-i (MCF7 cells stably expressing negative control for the inhibitor) and MCF7 130b-i (MCF7 cells stably expressing the oligonucleotide inhibitor of miR-130b-3p). Relative expression levels of α-SMA and SPIN90 of randomly selected ten areas were calculated using the ImageScope (Leica) software. Scale bar, 100 μm. **G** MCF7 cancer cells and HBF cells stably expressing miR-130b-3p inhibitor or negative control oligonucleotide were co-injected into the mammary fat pads of mice (*n* = 5). Tumor tissues were harvested 6 weeks later and weighed. All data are presented as mean ± standard deviation. *p ≤ 0.05; **p ≤ 0.01; ***p ≤ 0.001 (Student’s *t*-test).

We next asked whether miR-130b-3p increases with the development of luminal A breast cancer and contributes to the expression of SPIN90 in vivo. miR-130b-3p is evolutionarily conserved across mice and humans with a completely equivalent sequence. We thus injected human MCF7 cells into the mammary fat pads of 17ß-estradiol-supplemented NOD.Cg-*Prkdc*^*scid*^/J mice. RT-qPCR analysis of miR-130b-3p in plasma samples obtained from MCF7 cell-injected mice at various time points confirmed that the miR-130b-3p levels in mouse plasma increased in proportion to the tumor size (Fig. 5B). In addition, miR-130b-3p was markedly upregulated in RNA extracted from tumor tissues compared to homogenized normal adipose tissues (Fig. 5C).

To assess the expression patterns of miR-130b-3p and SPIN90, we performed both in situ hybridization (ISH) and immunohistochemistry (IHC) on xenografted tumors. Slide-mounted sections of tumor tissues (*n* = 4) from MCF7-injected mice were stained for ISH followed by IHC, and 10 randomly selected tumor or adjacent normal areas of each tissue were analyzed. Stromal fibroblasts in tumor and normal areas were distinguished by staining with antibodies to α-SMA and Vimentin [36], respectively. The fluorescence intensity of miR-130b-3p was obviously stronger than that of SPIN90 in tumor stroma but not in stroma from adjacent normal regions, which exhibited high-level expression of SPIN90 (Fig. 5D, E). To determine the CAF activation ability of miR-130b-3p in vivo, MCF7 cells expressing a nonspecific oligonucleotide (MCF7 NC-i) or a specific inhibitor sequence of miR-130b-3p (MCF7 130b-i) were injected into the mammary fat pads of mice, and the expression levels of α-SMA and SPIN90 were assessed by IHC of serial tumor tissues. The amount of α-SMA in the tumor stroma formed by MCF7 130b-i cells decreased compared to the control, whereas SPIN90 expression increased than control (Fig. 5F). In addition, co-injection of HBF cells expressing miR-130b-3p inhibitor and MCF7 cells reduced the growth of tumor tissue when compared with the control group (Fig. 5G). The data collectively support that miR-130b-3p is increased in luminal A breast cancer disease models, with increased expression of miR-130b-3p in breast CAFs being associated with reduced expression of SPIN90. Because miR-130b-3p does not affect the growth, migration, or invasion of MCF7 cancer cells (Supplementary Fig. S4A, B), these findings confirmed that miR-130b-3p inhibition significantly reduced the development of CAFs around cancer.

### miR-130b-3p exhibits a negative correlation with SPIN90 in human breast CAFs

**Fig. 6:**
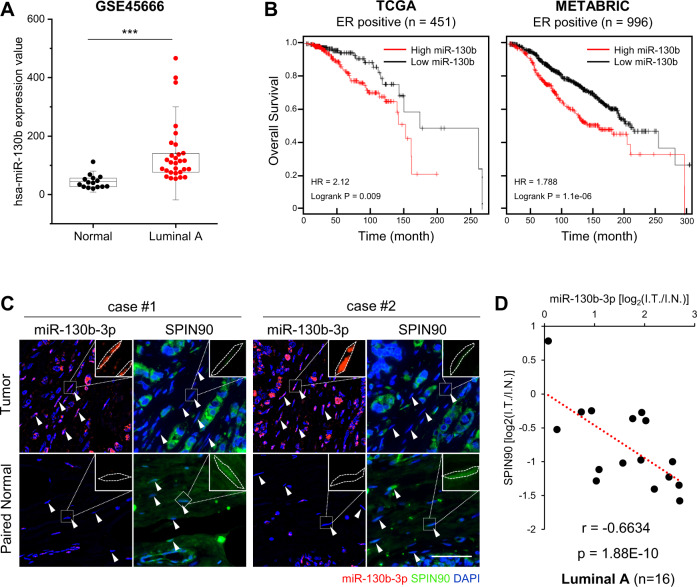
Expression of miR-130b-3p in CAFs derived from human breast cancer tissue is inversely correlated with that of SPIN90. **A** Expression of miR-130b in human luminal A breast cancer (*n* = 29) and normal tissues (*n* = 15) from the GSE45666 dataset. ***p ≤ 0.001 (Student’s *t*-test). **B** KM-plot survival analysis for ER-positive patients with respect to miR-130b expression. The data were retrieved from the TCGA (*n* = 451) and METABRIC (*n* = 996) datasets. HR hazard ratio. **C** Representative images of miR-130b-3p (red) and SPIN90 (green) staining in serial tissue sections from a tumor microarray (BR804b). Tumor and paired normal tissues from luminal A breast cancer patients (*n* = 16) were analyzed. The enlarged images at the upper right were not merged with DAPI. Cell boundaries are marked with white dotted lines. White arrows indicate stromal fibroblasts. Scale bar, 50 μm. **D** Correlation between the expression levels of miR-130b-3p and SPIN90 in the stroma. Each value was calculated as the log ratio of [intensity in tumor stroma] relative to [intensity in normal stroma] using ImageJ software.

We further examined the clinical significance of miR-130b-3p as a SPIN90 regulator in human data. To evaluate the expression of miR-130b in human breast tumor tissues, we retrieved miRNA expression data from GSE45666, which is a publicly available GEO dataset containing miRNA expression profiles of tissues from breast cancer patients and normal controls. Our analysis of these data revealed that miR-130b was upregulated 3.20-fold in luminal A-type breast tumor tissues (*n* = 29) compared to normal breast tissues (*n* = 15) with high significance (*p* = 0.000605, Fig. 6A). KM plot analysis for miR-130b using the TCGA and METABRIC breast cancer datasets revealed that the overall survival rate for patients with high-level miR-130b-3p expression was lower than that for those with low miR-130b among the ER-positive patients in all datasets (Fig. 6B).

To investigate the potential correlation between miR-130b-3p and SPIN90 in patients, we used ISH and IHC staining of serial human tumor microarray sections containing 16 paired samples representing luminal A breast cancer and paired normal breast tissues. As shown in Fig. 6C, miR-130b-3p was upregulated while SPIN90 was expressed at only a low level by CAFs near the tumor cells. In fibroblasts within paired-adjacent normal tissues, miR-130b-3p and SPIN90 presented trends opposite those seen in the tumor tissues. Taken together, our data collectively suggest that luminal A breast cancer patients with high-level miR-130b-3p expression show poor prognosis, and that miR-130b-3p shows an inverse correlation with SPIN90 in fibroblasts from human breast tumor tissues, with high significance (*p* = 1.88E−10, *r* = −0.6634) (Fig. 6D).

## Discussion

The TME does not simply constitute cancerous tissue, but rather actively communicates with cancer cells to promote cancer progression [37]. Fibroblasts, which are a key component of the TME, exhibit anti-tumorigenic behavior in the normal state but become a critical factor for cancer cell growth and therapeutic resistance upon tumor initiation [38]. Numerous studies have revealed that CAFs in breast cancer tissue not only promotes tumor progression but also contribute to metastasis by triggering ECM rearrangement and secreting paracrine factors [39]. Therefore, increasing studies are focusing on the search for inducers of CAF activation.

SPIN90 was originally identified as an adaptor protein that regulates actin polymerization and lamellipodium formation [40]. We recently reported on how SPIN90 modulates fibroblast activation and breast cancer. Depletion of SPIN90 in fibroblasts promotes microtubule stabilization and leads to the nuclear translocation of yes-associated protein for CAF activation during breast cancer progression [22]. Moreover, SPIN90 depletion enhances the expression of fibronectin containing an extra domain A and thereby causes ECM reorganization and tumor metastasis [33]. SPIN90 was reported to be downregulated in CAFs of breast cancer patients, and we herein show that this tendency is more pronounced in tissues of ER-positive patients.

In our efforts to elucidate the mechanism underlying the decrease of SPIN90 in breast CAFs, we focused on miRNAs. We demonstrated that miR-130b-3p binds to the 3’UTR of the *Spin90* mRNA, that miR-130b-3p activates fibroblasts, and that miR-130b-3p-activated fibroblasts promote the migration and invasion of breast cancer cells. The expression of *Spin90* mRNA and GFP reporter gene containing the 3’UTR of *Spin90* significantly reduced when miR-130b-3p mimic was transfected to HEK-293T cells. These results collectively suggest that miR-130b-3p has a seed region that is highly complementary to a region in the 3’UTR of the *Spin90* mRNA. The levels of expression of the top four genes predicted to have high binding scores for miR-130b-3p did not change following miR-130b-3p mimic treatment of HBF cells (Supplementary Fig. S1E). This indicated that miR-130b-3p should not have major off-target effects and that it mainly targets SPIN90 in HBF cells. We also showed that the ectopic expression of miR-130b-3p mimic in fibroblasts induced a decrease in SPIN90, efficiently increasing the levels of α-SMA (Fig. 3A), *Fap*, and *Ctgf* (Fig. 3B) thereby promoting fibroblast activation. Activated fibroblasts are known to be an indispensable factor for tissue fibrosis [41] and several studies have shown that miR-130b-3p is involved in fibrosis pathology [42, 43]. The TME has fibrotic tissue properties, wherein activated fibroblasts induce the deposition and reorganization of collagen [44]. Activated fibroblasts are divided into two main categories: myofibroblasts, which increase hypoxia, the stiffness of the ECM, and the invasion of tumor cells; and inflammatory fibroblasts, which affect tumor growth, immunosuppression, and stemness [45, 46]. We found that miR-130b-3p mimic-treated fibroblasts induce cancer cell migration and invasion. Therefore, the stroma of tumors enriched for miR-130b-3p will activate fibroblasts to myofibroblasts to form a collagen-rich TME in which cancer cells will become more active.

Our results demonstrated that miR-130b-3p is considerably expressed in most luminal A-type breast cancer tissues and cells. However, the expression of SPIN90, which is suggested to be a target of miR-130b-3p, was not downregulated in luminal A breast cancer cells (data not shown). We hypothesized that miR-130b-3p mainly exists as a precursor form in luminal A breast cancer cells, and is metabolized to the mature form after extracellular secretion. The miRNAs secreted from cancer cells are mainly delivered to the TME through exosomes [25]. Exosomes from cancer cells contain more Dicer than those from normal cells [47], meaning that cancer-cell-derived exosomes have a high ability to process pre-miRNA into mature miRNA. The relative expression levels of intracellular and secreted pre-miR-130b (Supplementary Fig. S2B, J, respectively) and the presence of Dicer in cancer cell CM (Supplementary Fig. S2I) collectively support the possibility that miR-130b-3p may undergo cell-independent processing in the TME. The reason that miR-130b-3p was detected in luminal A breast cancer cells at a level similar to the precursor form (Fig. 4C, Supplementary Fig. S2B) is presumed that mature miR-130b-3p is completely included in the precursor sequence, making it difficult to distinguish between the two forms using general RT-qPCR methods.

To verify whether miR-130b-3p increased in cancer cells is transported to CAFs through exosomes, we performed 10 kDa-centrifugal filtration of CM, because size-selective ultrafiltration-based methods have been shown to effectively enrich exosomes [48]. Our analysis of representative markers for exosomes and cytoplasm in the CM (Supplementary Fig. S2I) demonstrated that exosomal RNA was included in the enrichment process, whereas intracellular debris was not. The concentrated CM from luminal A breast cancer cells contained more miR-130b-3p than CM from normal epithelial cells, downregulated SPIN90 in fibroblasts, and activated fibroblasts (Fig. 4D, E). The tendency was reversed when CM of MCF7 cells with reduced miR-130b-3p (Supplementary Fig. S2K) was used. To clearly show that miR-130b-3p is secreted via exosomes from MCF7 cells, exosomes obtained by the ultracentrifugation of CMs of MCF10A and MCF7 cells were analyzed using transmission electron microscopy (TEM), nanoparticle tracking analysis, and western blotting of exosome markers (Supplementary Fig. S3A–C). MCF7 exosomes contained higher levels of miR-130b-3p than MCF10A exosomes (Supplementary Fig. S3D) and downregulated SPIN90 and upregulated α-SMA in fibroblasts (Supplementary Fig. S3E). Additionally, exosomes from MCF7 130b-i cells (miR-130b-3p-knocked down cells) were less effective at fibroblast activation than exosomes from MCF7 NC-i cells (Supplementary Fig. S3F–J). Furthermore, treatment of MCF7 cells with GW4869 to inhibit exosome formation [49] or treatment of MCF7 CM with RNase and Triton X-100 significantly reduced the amount of miR-130b-3p in MCF7 CM (Fig. 4F, Supplementary Fig. S2F, G). These findings indicate that miR-130b-3p is mainly secreted via exosomes from MCF7 cancer cells. MCF7 CM treated with miR-130b-3p inhibitor directly or CM of MCF7 with suppressed exosome formation failed to activate fibroblasts (Supplementary Fig. S2E, H), suggesting that exosomal miR-130b-3p regulates SPIN90 expression to activate fibroblasts directly.

miRNAs secreted from cancer cells often circulate in body fluids in a stable form; they can modulate the development of TME and are considered biomarkers of disease [50]. Examination of circulating miRNA is utilized for early diagnosis, prognosis, and therapeutic response prediction in breast cancer [51]. miR-21 and miR-196a are considered to be diagnostic and prognostic markers in breast cancer, respectively [52, 53], and miR-196a has been reported to be increased by estrogen to promote the growth of breast cancer cells [54]. However, little research has focused on identifying TME-modulating miRNAs that can be used as biomarkers [30]. Here, we confirmed that miR-130b-3p expression in plasma increased with tumor growth in mice that received fat-pad xenografts of luminal A cancer cells. Xenograft tumor sections exhibited increased miR-130b-3p in CAFs. In addition, a high level of miR-130b-3p in tissues predicted poor outcomes in luminal A-type breast cancer patients. Taken together, our present findings validate the potential of miR-130b-3p as a biomarker of luminal A breast cancer progression and show the effect of miR-130b-3p in the breast TME.

Dysregulation of miR-130b-3p has been reported in various types of cancer, but the mechanism underlying the modulation of miR-130b-3p expression in breast cancer remains unknown. miRNA expression is known to be regulated by DNA methylation, transcription factors, and defects in the miRNA biogenesis machinery [27]. We performed experiments involving 5-aza-2’-deoxylcytidine treatment and methylation-specific PCR assays but did not find evidence that DNA methylation changes modulate miR-130b-3p expression in our system (data not shown). As shown in Fig. 4C and Supplementary Fig. S2B, the mature and precursor forms of miR-130b-3p in luminal A breast cancer were endogenously enhanced compared to the levels seen in other breast cancer types, indicating that the expression of this miRNA is specific to the luminal type. The gene promoter characteristics and transcription factors essential for miRNA expression differ according to the breast cancer subtype [55]. Several transcription factor candidates that might be involved in the transcription of genes containing miR-130b-3p were predicted using the bioinformatics tool, TransmiR (http://cmbi.bjmu.edu.cn/transmir). Our preliminary analysis indicates that these transcription factors are higher in MCF7 cells, but not in normal cells, MCF10A and in triple-negative breast cancer (TNBC) cells, MDA-MB-231 (data not shown). Further experimental validation is warranted to address this open question.

In sum, we herein propose that miR-130b-3p is a suppressor of SPIN90 during CAF activation in the breast TME. In the stroma of human luminal A breast cancer patient tissues, miR-130b-3p shows an inverse correlation with SPIN90, clinically supporting our other findings. Although 74.0% of luminal A type breast cancer patients survive to the end of a nearly 10-year follow-up [56], the distant recurrence rate in ER-positive patients who had 5-year course of chemotherapy is 1.4–1.8% in annual, and the risk approaches 21% at year 20 even at T1N0 state [57]. Distal tumor recurrence and metastasis are highly promoted by CAFs through their ability to develop a niche for cancer cells [8]. Therefore, clinical trials targeting CAFs to improve cancer outcomes have recently emerged [58]. We herein demonstrate that miR-130b-3p is mainly derived from luminal A breast cancer cells and functions to activate CAFs, which subsequently increase cancer cell invasiveness. Both transient and stable transfections of miR-130b-3p oligonucleotide inhibitors in vitro and in vivo returned the miR-130b-3p-activated CAFs to a normal state, emphasizing the importance of the miR-130b-3p/SPIN90 axis for CAF activation and supporting the potential for this oligonucleotide to act as a therapeutic inhibitor of CAFs in breast cancer.

## Materials and methods

### Human tissue and data

Breast cancer with matched adjacent normal breast tissue arrays (BR804b; 40 cases, 80 cores, 1.5 mm diameter, 5 μm thickness) including information about luminal subtypes were purchased from US Biomax (Derwood, MD, USA). The miRNA expression profile data (GSE45666) was obtained from Gene Expression Omnibus (https://www.ncbi.nlm.nih.gov/geo/query/acc.cgi?acc=GSE45666). The GSE45666 dataset was based on the platform GPL14767 (Agilent-021827 Human miRNA Microarray G4470C) and contained 101 breast cancer and 15 normal breast tissue samples. The data derived from 29 luminal A breast cancer patients were applied to analysis to compare miR-130b expression with that of normal control groups.

### Cell culture

Human breast skin fibroblast (HBF, CCD1087sk) was purchased from ATCC (Manassas, VA, USA). MCF7 was kindly provided by Dr. E.K. Lee (Catholic University, Seoul, Korea). T47D, BT-474, and MDA-MB-231 were given favor by Dr. J.S. Nam (GIST, Gwangju, Korea). All cell lines except for MCF10A were cultured in Dulbecco’s modified Eagle’s medium (DMEM, high glucose; Gibco, Billings, MT, USA) supplemented with 10% fetal bovine serum (FBS), 100 unit/mL penicillin, 100 μg/mL streptomycin, and 250 ng/mL amphotericin B (Gibco) in a humidified incubator at 37 °C and 5% CO_2_. MCF10A was cultured in DMEM/F-12 (Gibco) containing 20 ng/mL EGF, 500 ng/mL hydrocortisone, 100 ng/mL cholera toxin, 10 μg/mL insulin, and antibiotics. All cell lines were confirmed to be free of mycoplasma using e-Myco VALiD Mycoplasma PCR Detection kit (iNtRON, Seoul, Korea).

### Cell transfection

To generate GFP reporter gene construct, *Spin90* mRNA 3’UTR (700 nt) was amplified from the total cDNA of HEK-293T cells using PrimeSTAR HS Polymerase (Takara, Kyoto, Japan) and the primers 5’-GCTCAAGCTTCGAATTCCTAGCACCTTGCTGTCCTCC-3’ (forward) and 5’-CCGCGGTACCGTCGACTTTCCAGATGCCTGTGATTT-3’ (reverse). The seed region of miR-130b-3p of *Spin90* 3’UTR was substitution mutated using the primers 5’-CCCTAAGAATGTTACGAGTTGACAGTGGGAGGGG-3’ (forward) and 5’-CCCCTCCCACTGTCAACTCGTAACATTCTTAGGG-3’ (reverse). The vector pEGFP-C1 was digested with the restriction enzymes EcoRI and SalI (NEB, Ipswich, MA, USA), and the amplicons were inserted into the vector using an EZ-Fusion Cloning Kit (Enzynomics, Daejeon, Korea). The cell lines were treated using Dharmafect 1 (Dharmacon, Lafayette, CO, USA) for transfection of miR-130b-3p mimic (Dharmacon), miRCURY LNA miR-130b-3p power inhibitor (QIAGEN, Hilden, Germany), and Lipofectamine 3000 (Invitrogen, Waltham, MA, USA) for transfection of DNA constructs according to the manufacturer’s instructions. Lentiviral vector for anti-miR-130b-3p (HmiR-AN0159-AM04; GeneCopoeia, Rockville, MD, USA) was transfected to MCF7 and selected using 0.1 mg/mL hygromycin. The medium was replaced by DMEM with 10% FBS after 6 h of transfection, and cells were collected for analysis after 48 h.

### RT-qPCR

Total RNAs including miRNAs from cell lines, plasma, and frozen tissues were extracted using RNeasy Micro kit (QIAGEN) following the manufacturer’s protocol. Complementary DNAs (cDNAs) for miRNAs and mRNAs were synthesized by miScript II RT kit (QIAGEN) and TOPscript RT DryMIX (Enzynomics), respectively. Relative expression of the gene was measured by real-time-quantitative PCR on LightCycler 480 System (Roche, Basel, Switzerland) with miScript SYBR Green PCR kit (QIAGEN) or TB Green Premix Ex Taq (Takara). Detailed primers used are listed as follows; *spin90*: 5’-GCAGCCATCATCTCCACGCT-3’ (forward), 5’-ATGGCCAGGATGAGGGCAGA-3’ (reverse); *fap*: 5’-CCAGAATGTTTCGGTCCTGT-3’ (forward), 5’-CGAAATGGCATCATAGCTGA-3’ (reverse); *ctgf*: 5’-CTTGCGAAGCTGACCTGGAAGA-3’ (forward), 5’-CCGTCGGTACATACTCCACAGA-3’ (reverse); *gapdh*: 5’-GTCTCCTCTGACTTCAACAGCG-3’ (forward), 5’-ACCACCCTGTTGCTGTAGCCAA-3’ (reverse). Primers for hsa-miR-130b-3p and hsa-miR-130b were purchased from QIAGEN, as miScript primer assays.

### Western blotting

Washed cells were lysed with RIPA buffer (50 mM Tris–HCl, pH 7.4, 150 mM NaCl, 1% NP-40, 0.1% SDS) containing 10 mM NaF, 1 mM Na_3_VO_4_ and protease inhibitor cocktail (Roche). After electrophoresis and transfer process, the blots were incubated with antibodies against SPIN90 (clone 84B3, lab-made), α-SMA (14968, CST, Danvers, MA, USA), α-tubulin (clone DM1A, T6199, Sigma, St. Louis, MO, USA), Dicer (clone 13D6, ab14601, Abcam, Cambridge, UK), GFP (lab-made), and Exosomal Marker Antibody Sampler Kit (74220, CST).

### Cancer cell migration, invasion assay

MCF7 cells were seeded on culture-insert 2 well (ibidi, Gräfelfing, Germany) at 5 × 10^5^ cells/mL density, 70 μL for each well for wound healing assay. After 18 h, the insert was gently removed, and 1 mL of conditioned media incubated with miRNA-transfected HBFs for 25 h was treated on a confluent MCF7 cell layer. Gap closure was measured after 36 h under an inverted microscope.

For the modified Boyden chamber assay, 5 × 10^4^ of MCF7 cells were plated on 8.0 μm-pore transwell coated with or without 3.33% Matrigel (Corning, NY, USA). Conditioned media derived from miRNA mimic transfected HBFs was added to the lower chamber, and incubated for 25 h. Migrated or invaded cells were fixed and stained using 4% paraformaldehyde and 0.05% crystal violet, respectively, then counted in 9 randomly selected areas under an inverted microscope. All images were analyzed using ImageJ software.

### Exosomal miRNA collection and characterization

MCF10A, MCF7, T47D cells were plated at 80% confluency on 150 mm culture dishes. Cells were washed with DMEM serum-free media, then incubated in DMEM for 48 h. CM was centrifuged at 300 × *g* for 10 min and filtered through 0.2 μm syringe filter (Sartorius, Göttingen, Germany). Filtered CM was loaded on PBS-equilibrated Pierce protein concentrator (10 K MWKO; Thermo Scientific), and centrifuged at 4000 × *g*, 4 °C continuously till all CM are concentrated more than 10 times. For buffer change, 20 mL of PBS was added to the filter, and additional centrifugation was performed until the concentrated CM volume reached 300 μL. To harvest exosomes secreted from the cells, CMs were centrifuged at 4 °C for 10 min at 300 × *g*, 20 min at 16,500 × *g*, and 2 h at 200,000 × *g* [59, 60]. The collected exosomes were observed using a transmission electron microscope (JEOL, Tokyo, Japan) at the GIST Central Research Facility and quantified using Nanosight NS300 (Malvern Panalytical, Malvern, UK) at the Yoon Idea Lab (Seoul, Korea).

### Immunohistochemistry

Sections of tumor microarray and breast cancer tissue resected from mice were deparaffinized and rehydrated in routine series. Antigen retrieval was performed with IHC-Tek epitope retrieval solution (IHC World, Woodstock, MD, USA) in a humidified-heated chamber. Sections were incubated overnight at 4 °C with antibodies to SPIN90 (lab-made), α-SMA (CST), Vimentin (clone V9, sc-6260, Santa Cruz, Dallas, TX, USA), and E-cadherin (clone G10, sc-88426, Santa Cruz) overnight at 4 °C. The nuclei were counterstained with DAPI or Mayer’s hematoxylin (Dako, Santa Clara, USA), and the stained area was observed using a confocal microscope (FV1000; Olympus, Tokyo, Japan), research slide scanner (Olympus), or Aperio ImageScope (Leica, Wetzlar, Germany).

### In situ hybridization

Deparaffinized and rehydrated tissue sections were treated with 3% H_2_O_2_ (Sigma-Aldrich, St. Louis, MO, USA) to inhibit intrinsic peroxidase activity. After antigen retrieval in a heated chamber, the sections were incubated with locked nucleic acid (LNA)-modified oligonucleotide probe for hsa-miR-130b-3p (QIAGEN) at 54 °C, for 1 h. Immediately after, a stringent wash was performed in 54 °C-heated SSC buffer (Sigma-Aldrich) according to the manufacturer’s protocol. Samples were incubated with a 1:400 dilution of anti-DIG-POD antibody (11207733910, Roche), and treated with TSA-plus Cy3 amplification solution (Akoya Biosciences, Marlborough, MA, USA). Amplified fluorescence signals were detected by confocal microscopy (FV1000), or research slide scanner (Olympus).

### Estradiol supplement and breast tumor xenograft

9-to-12-week-old female NOD.Cg-*Prkdc*^*scid*^/J mice were used in this study. To supply mice with estradiol for xenograft luminal A breast cancer cells, 10 mg/mL of 17β-estradiol (Sigma-Aldrich) in sesame oil (Sigma-Aldrich) was filled into silastic capsules (inner/outer diameter/length: 2.0/3.0/10.0 mm). The estradiol capsules were incubated overnight in a conical tube containing the same solution as the capsules, and inserted subcutaneously in the dorsal neck of the anesthetized mice [61]. A week later, a breast tumor xenograft was performed. 2 × 10^6^ cells of MCF7 or HBF suspended in 1:1 of matrigel:DMEM were injected into the left fifth mammary fat pad. Tumor volume was estimated with this formula: volume = (length × width^2^)/2.

### Statistical analysis

All experiments were carried out in triplicate, and data on the graph were presented as mean ± standard deviation. The correlation between miR-130b-3p and SPIN90 was evaluated by Pearson’s test. A two-tailed Student’s *t*-test was used to assess the *p*-value, and *p ≤ 0.05, **p ≤ 0.01, ***p ≤ 0.001 were considered statistically significant.

## Supplementary information

Supplementary Fig. S1-4

## Data Availability

The data generated during the current study are available from the corresponding author on reasonable request.
