# Supplementary material for: Tumor-derived miR-130b-3p induces cancer-associated fibroblast activation by targeting SPIN90 in luminal A breast cancer
Source: Oncogenesis. 2022 Aug 10;11(1):47. doi: 10.1038/s41389-022-00422-6 (PMC9365846; doi:10.1038/s41389-022-00422-6)
Supplement: Supplementary file 1 — Supplementary Fig. S1-4 [file 41389_2022_422_MOESM1_ESM.pdf]

Supplementary Fig. S1

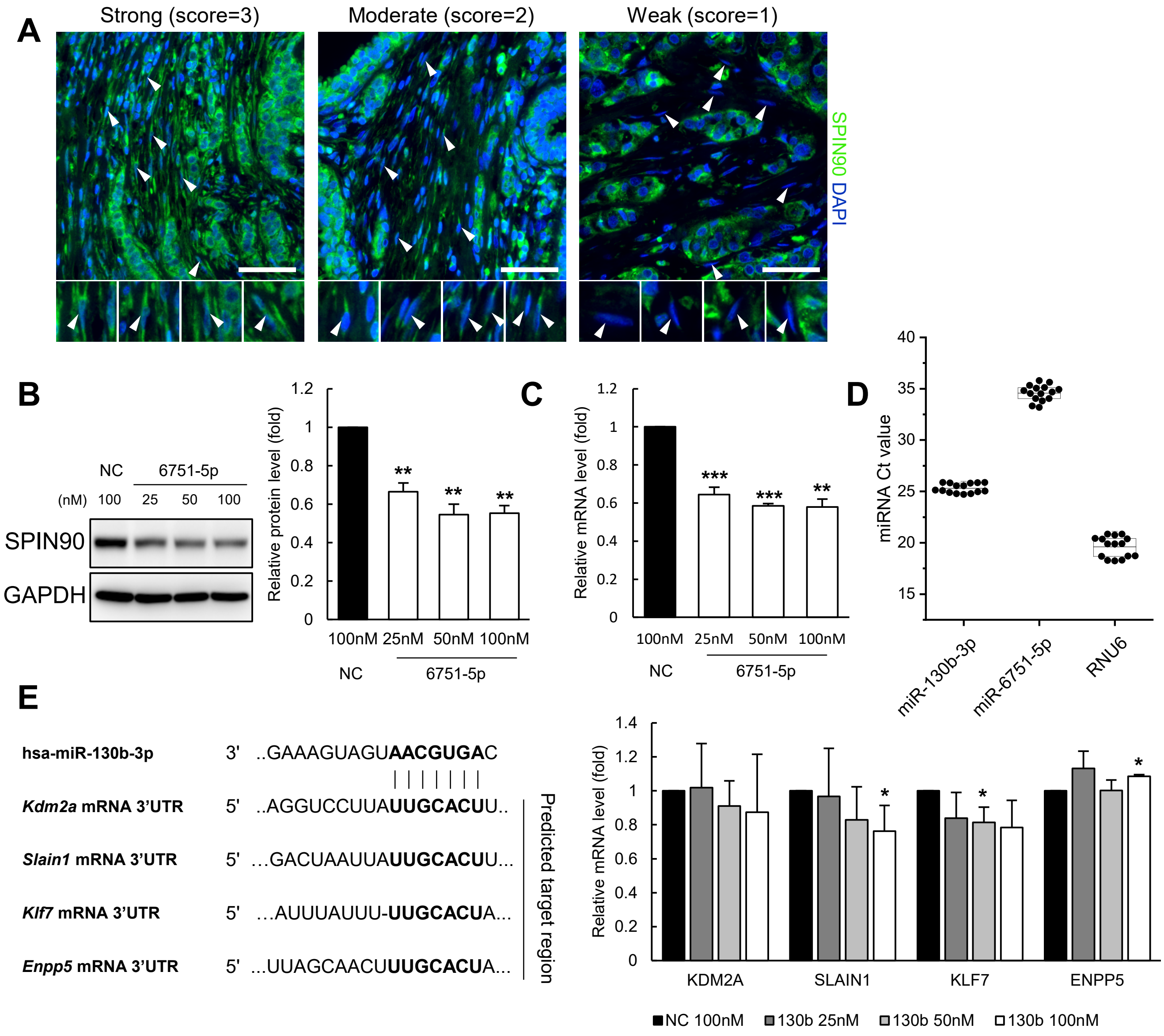

Supplementary Fig. S1 Selection process of SPIN90-targeting miRNA in the range of ER-positive breast cancer. **A**, Representative images for classification criteria (strong=3; moderate=2; weak=1) of SPIN90 expression in tumor stroma. White arrows, stromal fibroblasts. Scale bar, 50 μm. **B**, SPIN90 protein downregulation by another SPIN90-targeting candidate miRNA, miR-6751-5p. Here, the indicated concentration of miR-6751-5p was transfected to HEK-293T cells for 48 h, and Western blotting data were normalized to the expression level in the negative control (n=3). **C**, HEK-293T cells were transfected with miR-6751-5p mimic, and the *Spin90* mRNA level was quantified by RT-qPCR assay (n=3). **D**, Comparison of miRNA Ct values between miR-130b-3p and miR-6751-5p in MCF7 cells. RNU6 was used as an endogenous control. **E**, Confirmation that miR-130b-3p mainly targets genes other than SPIN90 in HBFs. The top four genes predicted by TargetScan to bind miR-130b-3p are listed. Bolded sequences indicate the predicted target region. Expression levels of the predicted genes were quantified by RT-qPCR after miR-130b-3p mimic transfection (n=3). All data are presented as mean ± standard deviation. *$p \leq 0.05$; **$p \leq 0.01$; ***$p \leq 0.001$ (Student's *t*-test).

Supplementary Fig. S2

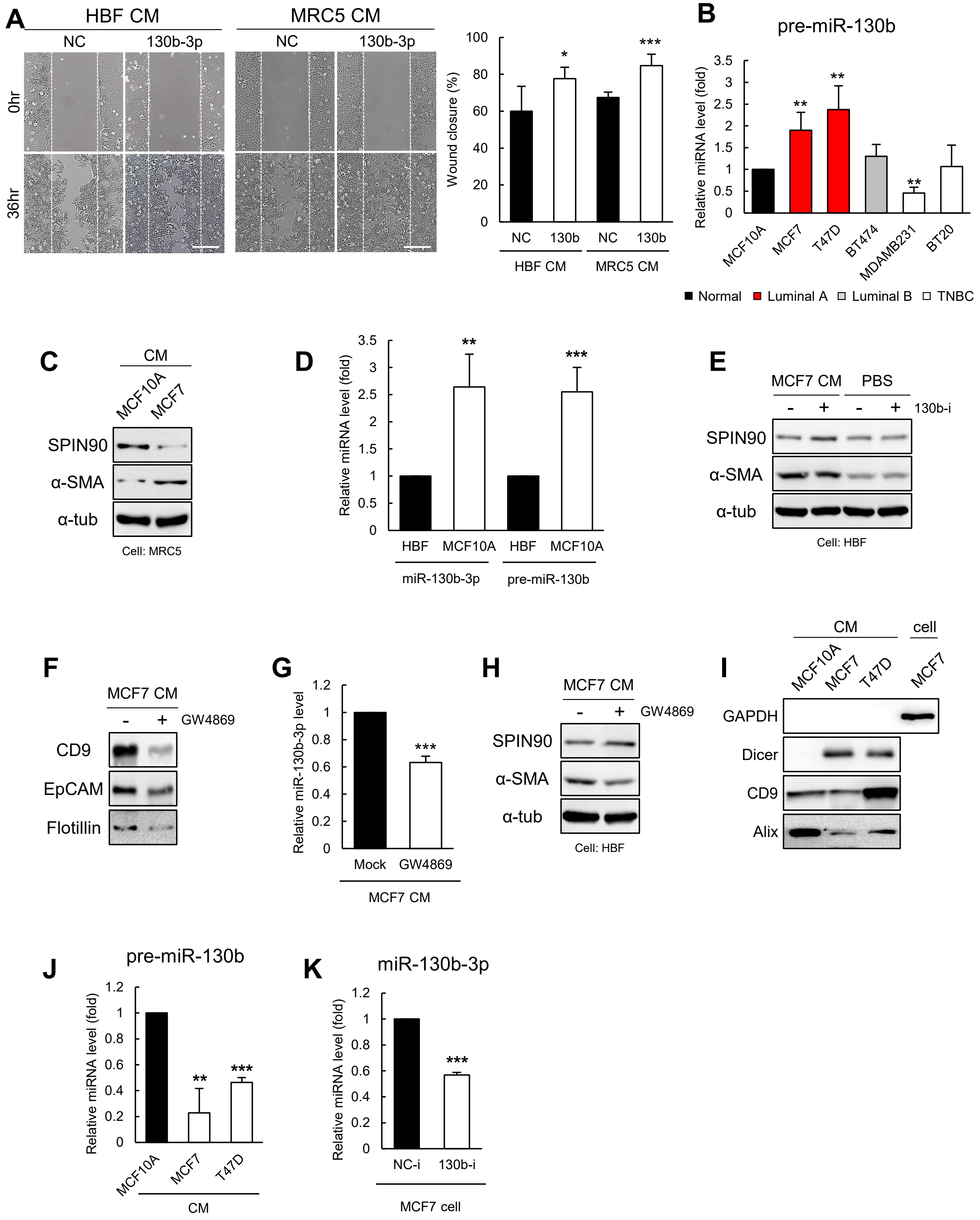

Supplementary Fig. S2

Supplementary Fig. S2 Confirmation of the effect of miR-130b-3p and its transport to fibroblasts. A, Wound-healing assay of MCF7 cells. HBFs and MRC5 cells were transfected with miRNAs for 48 h, serum-free medium was added, and CM was collected. Scratch-wounded MCF7 cell monolayers were incubated with CM for 36 h. Scale bar, 100 μm. **B**, RT-qPCR analysis quantified the expression of precursor miR-130b in cancer cell lines of various luminal types, compared to that in normal MCF-10A cells (n=3). The level of each miRNA was normalized to that of RNU6. **C**, Western blot analysis of fibroblast activation and SPIN90 downregulation in MRC5 cells following incubation with MCF7 CM. CM from MCF10A cells was regarded as a control. **D**, RT-qPCR assay examining the endogenous levels of miR-130b-3p and pre-miR-130b in HBFs compared to MCF10A cells (n=3). **E**, Western blot analysis of HBF incubated with CM of MCF7 cells, or PBS, transfected with an oligonucleotide inhibitor of miR-130b-3p. Buffer was replaced in all groups to eliminate free miR-130b-3p inhibitor and residual Lipofectamine 3000. **F**, Western blot analysis of the expression of exosome markers in CM of MCF7 in the presence or absence of 10 μM GW4869, a representative inhibitor of nSMase, a protein involved in exosome formation. **G**, Relative miR-130b-3p levels in the CM of GW4869-treated MCF7, as quantified by RT-qPCR assays. **H**, HBF cells were treated with CM derived from GW4869-treated MCF7 cells to confirm the effect of cancer-derived exosomes on fibroblast activation. **I**, The levels of CD9, Alix (exosome marker), GAPDH (cytoplasm marker), and Dicer in concentrated CM were assessed through Western blotting. **J**, Expression of precursor miR-130b-3p in concentrated CM (n=3). **K**, miR-130b-3p level in MCF7 cells stably expressing an inhibitor of miR-130b-3p (n=3). All data are presented as mean ± standard deviation. *$p \leq 0.05$; **$p \leq 0.01$; ***$p \leq 0.001$ (Student's *t*-test).

Supplementary Fig. S3

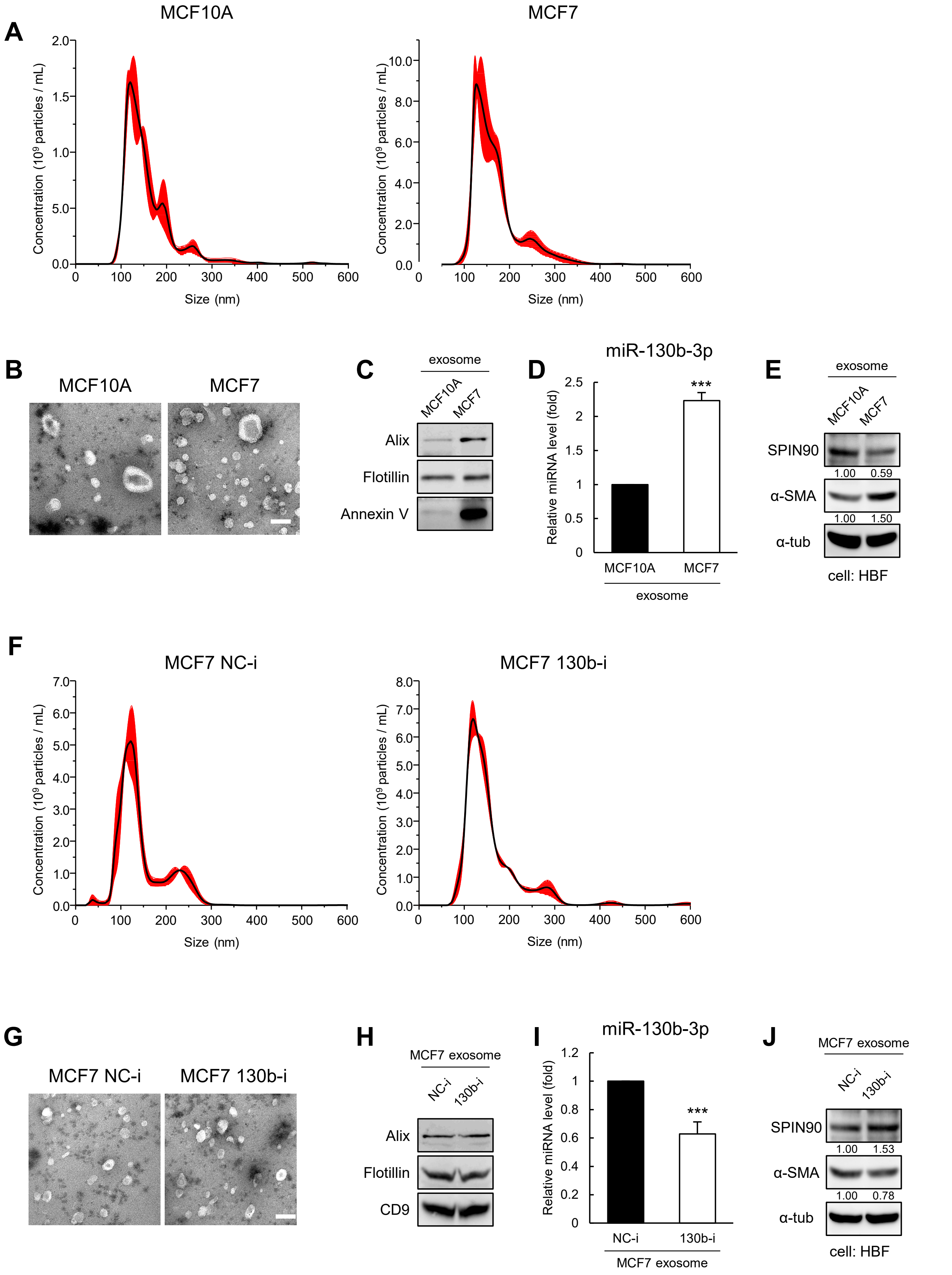

Supplementary Fig. S3

Supplementary Fig. S3 Confirmation of transport of miR-130b-3p-containing exosome from MCF7 to fibroblasts. **A**, **B**, Exosomes secreted from MCF10A and MCF7 cells detected by transmission electron microscopy (TEM) and nanoparticle tracking analysis. Scale bar, 100 nm. **C**, Expression of representative exosome markers, Alix, Flotillin, and Annexin V, in exosomes derived from MCF10A and MCF7 cells assessed by western blotting. **D**, Expression of miR-130b-3p in exosomes derived from MCF10A and MCF7 cells analyzed by RT-qPCR. **E**, Fibroblast activation ability of MCF7 exosomes assessed by western blotting. HBFs were treated with exosomes derived from MCF10A and MCF7 cells for 48 h and collected. **F**, **G**, Exosomes secreted from MCF7 NC-i and MCF7 130b-i cells detected by TEM and nanoparticle tracking analysis. Scale bar, 100 nm. **H**, Expression of representative exosome markers, Alix, Flotillin, and CD9, in exosomes derived from MCF7 NC-i and MCF7 130b-i cells assessed by western blotting. **I**, Expression of miR-130b-3p in exosomes derived from MCF7 NC-i and MCF7 130b-i cells analyzed by RT-qPCR. **J**, Fibroblast activation ability of MCF7 130b-i exosomes assessed by western blotting. All data are presented as mean ± standard deviation. ***$p \leq 0.001$ (Student's *t*-test).

Supplementary Fig. S4

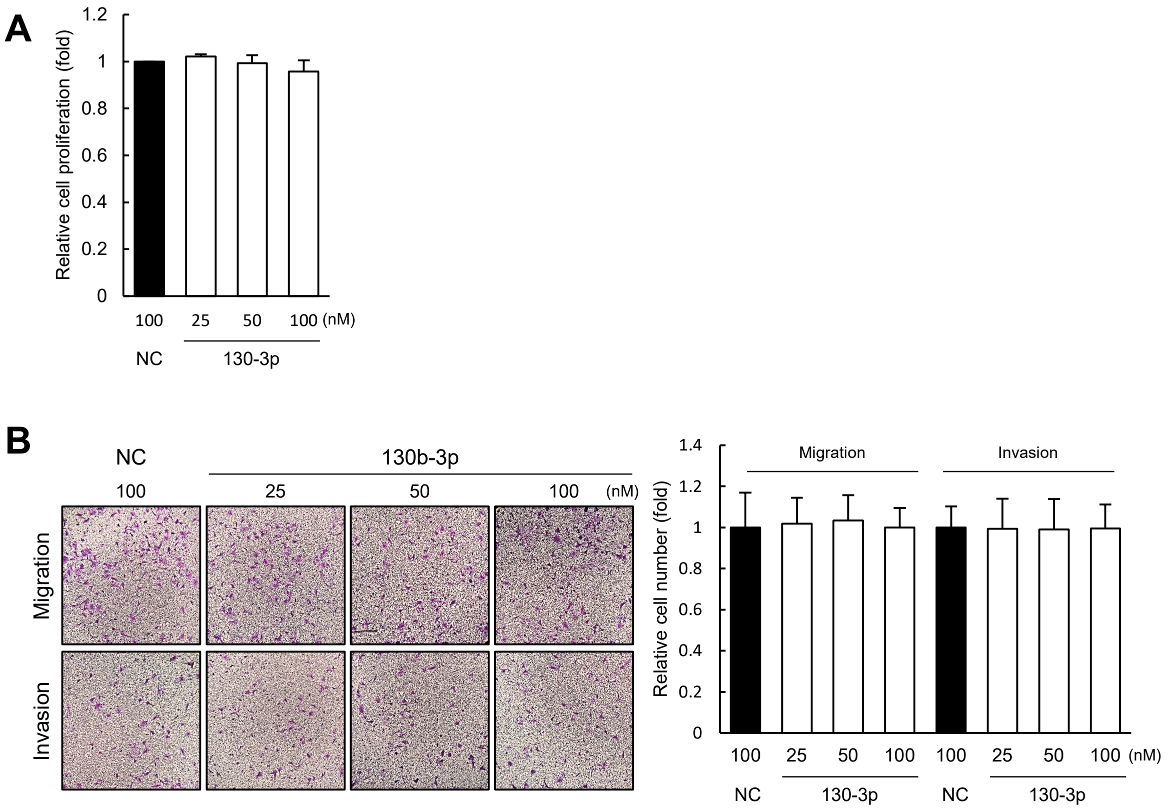

Supplementary Fig. S4 Effect of miR-130b-3p on MCF7 cancer cell growth, migration, and invasion. A, Relative cell proliferation rates of miR-130b-3p transfected MCF7 cancer cells. Cells were transfected with various concentrations of miR-130b-3p and seeded 48 h later into 96 well plates for MTT assays. **B**, Migration and Invasive ability of miR-130b-3p transfected MCF7 cells. Images of migrating and invading cells were taken 24 h after seeding in a Boyden chamber, with the images analyzed using ImageJ software.
